# Impact of Workplace Conflicts on Self-Reported Medically Certified Sickness Absence in Latvia

**DOI:** 10.3390/ijerph18031193

**Published:** 2021-01-29

**Authors:** Svetlana Lakiša, Linda Matisāne, Inese Gobiņa, Ivars Vanadziņš, Lāsma Akūlova, Maija Eglīte, Linda Paegle

**Affiliations:** 1 Institute for Occupational Safety and Environmental Health, Rīga Stradiņš University, Dzirciema 16, LV-1007 Rīga, Latvia; Linda.Matisane@rsu.lv (L.M.); Ivars.vanadzins@rsu.lv (I.V.); Lasma.Akulova@rsu.lv (L.A.); Maija.Eglite@rsu.lv (M.E.); Linda.Paegle@rsu.lv (L.P.); 2 Department of Public Health and Epidemiology, Rīga Stradiņš University, Kronvalda Boulevard 9, LV-1010 Rīga, Latvia; Inese.Gobina@rsu.lv; 3 Institute of Public Health, Rīga Stradiņš University, Kapseļu Street 23, LV-1046 Rīga, Latvia

**Keywords:** psychosocial risk factors, stress at work, conflicts at work, sick leave, sickness absence, labour absenteeism

## Abstract

Sickness absence is one of the most important working population health indicators. It is a complex phenomenon that is investigated by health care and occupational health specialists, economists, and work psychologists. Sickness absence is used as a predictor for morbidity and mortality, but besides the health status of an individual, sickness absence is influenced by demographic, socio-economic factors, and work environment factors. Conflicts at work are a common psychosocial risk factor that can affect sickness absence. The aim of the study was to investigate the association between different types of workplace conflict and self-reported medically certified sickness absence using cross-sectional survey data pooled from four periodic national surveys—Work conditions and risks in Latvia (2006–2018). The sample is representative of the working population of Latvia, as respondents were randomly drawn from different regions and industries. In total, the study sample (*n* = 8557) consisted of employees between 16 and 80 years old (average 42.8 +/− 12.6) of which 46.2% were males and 53.8% were females. Researchers used the computer-assisted personal interviewing (CAPI) method for collecting data. The association between workplace conflicts and sickness absence was analysed by using binomial logistic regression and calculated as odds ratios (OR) with 95% confidence intervals (CI), with adjustment for gender, age, education and survey year. The risk of sickness absence was higher among women (OR = 1.24, CI 1.13–1.35), employees aged 25–44 years old and employees with higher income. Controlling for socio-demographic factors and survey year, the odds of sickness absence increased significantly for all types of workplace conflict analysed. The strongest association with sickness absence was related to conflicts between managers and employees (OR = 1.51, CI 1.37–1.66) and conflicts between groups of employees (OR = 1.45, CI 1.31–1.61). Conflicts between employees and with customers also increased the odds of sickness absence (OR = 1.39, CI 1.27–1.52 and OR = 1.11, CI 1.01–1.23, respectively). Our findings suggest that tailored interventions at a company level for reducing workplace conflicts as risk factors of sickness absence are required. Those should focus on the improvement of managers’ leadership and human resource management skills.

## 1. Introduction

The costs related to sickness absence are a substantial burden to employers, governments and sick employees themselves. The Employers’ Confederation of Latvia has highlighted the problem in relation to the increasing numbers and costs of sickness absence in Latvia. Long term data on employees’ sickness absence from national registers in Latvia are not available in full. This is due to the fact that, until 2018, sick leave was issued by general practitioners and sickness benefits were partly paid by the employer without any centralised reporting. Since the beginning of 2018, a new e-health platform has been introduced that requires general practitioners to issue sick leave through its online system. Data from the new e-system show an almost 5% increase in the number of sick leave instances issued in 2019 compared to 2018. In addition, the number of sick days as part of these medically certified instances of sick leave has increased to more than 667,000 days within just one year [1]. These results suggest that sickness absence is an increasing burden on the state and employers in Latvia and requires action.

The official statistics on medically certified sick leave does not include information on such individual factors like education which has been documented to affect sickness absence through different pattern, e.g., knowledge on health behaviours, lifestyle, work-related risk factors, socioeconomic differences, etc. [2]. Individual factors related to sickness absence have been addressed in the Work conditions and risks in Latvia national survey which has been conducted on a regular basis since 2006. Previously published results show that the prevalence of self-reported medically certified sickness absence has increased slightly over the years with the exception of 2010. This has been mainly explained with the after effects of the financial crisis—this caused fear of job loss among employees should they take sick leave, less social security due to the lively shadow economy, amendments to the Law on Sickness Insurance and changes in how the state calculated social insurance benefits [3,4,5,6].

Although, by definition, sickness absence is an absence from work due to personal illness, it is in fact a more complex phenomenon [7]. An increasing amount of international research links the frequency and length of sickness absence to a range of different factors, including those related to individual health and psychological and social workplace risk factors. In general, the following sickness absence predictors have been identified: social factors (e.g., national social security system including sickness absence compensation, health care, sickness absence approval/certification system, absence culture) [8,9], work related factors (e.g., role at work, work conditions, salary, job demands in terms of workload, role conflict, work pace) [8,9,10,11,12,13,14,15,16,17], organisational factors (e.g., company size, the existence of health promotion programs, absence and/or promotion policies, quality of leadership and feedback, team climate) [8,9,10,11,12,13,14,15,16,17], family factors (e.g., marital status, problems in family, work–life balance) [8,18,19], and individual factors (e.g., sex, age, personality, education level, health, perception of health, desire to extend a weekend or holiday, job satisfaction, emotional dissonance, sleep length and sleep disturbances) [7,8,9,11,12,13,16,20,21,22,23].

Sickness absence is not a direct indicator of physical health, but it is associated with an employee’s motivation to go to work (e.g., a medical condition could make it impossible to go to work, but in most cases the individual has some degree of choice) [24,25]. In addition, existing evidence suggests that the causes for short-term and long-term sickness absence might differ. Long-term sickness absence presents the existence of objective health problems, but short-term sickness absence is more associated with other individual-related factors like how one copes with a poor psychosocial environment at work, low levels of motivation, satisfaction and commitment, and the desire to extend a weekend or holiday [21,26,27].

Conflicts at work may influence sickness absence among employees through different processes. A decrease in individual well-being, which can be moderated by personality, leading to sickness absence can be observed through poor mental health, strain, burnout, depression, anxiety, psychiatric morbidity, etc. [28,29]. If sickness absence is not completely explained by the employee’s state of health, it might be at least to some extent explained by reduced well-being and a poor psychosocial environment at work. The occurrence of workplace conflict is unavoidable as it is a part of everyday business life; however, it is manageable [30,31]. This opens a wide perspective for employers, occupational health and safety professionals, as well as state institutions in the area of targeted prevention activities, to improve the psychosocial working environment [32]. It appears that sickness absenteeism caused by different types of workplace conflict creates a major challenge for leadership and human resource management [33].

When analysing individual effects of workplace conflicts, research mainly focuses on mental health, job satisfaction, stress, psychosomatic symptoms, burnout, etc., as an outcome, but sickness absence, which is the focus of our study, has been less studied [34]. Our study tries to address the research gap by assessing the association between different types of workplace conflict and self–reported medically certified sickness absence by adjusting for socio-demographic factors and survey year among employees in Latvia.

## 2. Materials and Methods

### 2.1. Study Population and Sample

The Work conditions and risks in Latvia national surveys were conducted in Latvia in 2006 [3], 2010 [4], 2013 [5] and 2018 [6]. The aim of these surveys was to gather evidence on situations related to occupational risk factors, occupational health, safety and official employment statuses that would serve as a basis for effective decision making in the creation and adjustment of employment and social policy programs to ensure sustainable development. A cross-sectional study design was used to examine the association between conflicts at work and self-reported medically certified sickness absence. Only employees who had either reported being sick within the previous year and had taken medically certified sickness absence for their illness or reported not being ill in the previous year were included in the data analysis. The number of respondents per each survey year is described in Table 1.

The original study population consisted of respondents from different groups within the working population—employees with official contracts with one or more employers, the self-employed, “pseudo” self-employed, persons on maternity leave, etc. Our analysis considered only employees to narrow the study population and obtain a homogenous study sample.

The study sample is representative of the working population in Latvia as the 8557 employees were randomly drawn from different regions and industries. The average age of respondents was 42.8 +/− 12.6 (min 16, max 80 years), 46.2% were males and 53.8% females (Table A1).

A combined sampling method—quota and stratified random sampling—was used to recruit the study population. The interviewers used the Computer-Assisted Personal Interviews (CAPI) method to collect data at respondents’ homes. For data comparison purposes, the questions and answers used in the survey interviews have remained unchanged throughout the years.

### 2.2. Study Variables

#### 2.2.1. Outcome Variable

The outcome variable of this study is self-reported medically certified sickness absence (referred to as sickness absence hereinafter) within the previous year which was measured by the question “Which of the following situations regarding ill health within the previous year apply to you personally?”. Several answers were possible: “I was ill and took medically certified sickness absence”; “I was ill but did not take medically certified sickness absence”; “I was ill but I went to work (worked) while being ill”; “I was not ill within the previous year”. 

Respondents who answered “I was ill but did not take medically certified sickness absence” were excluded from the analysis as evidence suggests that self-certified sickness absence may represent a different group of underlying causes for sickness absence [26,35,36]. In addition, the legal system in Latvia does not allow employees to take self-certified sickness absence. If self-certified sickness absences do occur, these situations are individual agreements between an employer and an employee or, in rare cases, are agreed at a company level. Respondents who answered “I was ill but I went to work (worked) while being ill” were considered to be sickness presenters and were also excluded from the analysis. Respondents reported “I don’t know/hard to say” were considered as missing values and also excluded.

Two groups of respondents were created for data analysis purposes: (1) employees who reported being sick within the previous year and had taken medically certified sickness absence and (2) employees who reported not being ill within the previous year.

#### 2.2.2. Independent Variables

Different types of conflict at the workplace were analysed as independent factors in association with sickness absence. During the interview, respondents were asked: “Please specify how often the following situations occur in your workplace: … conflicts between management/supervisors and employees, conflicts with other employees, conflicts within groups of employees and conflicts with customers”. The difference between the answers to “conflicts with other employees” and “conflicts within groups of employees” was the number of people involved in the conflict. The first answer applied to when the conflict happened between individuals while the second one applied to when the conflict happened in groups. 

To evaluate the frequency of conflicts, the following Likert scale answers were used: “rather often”, “sometimes”, “rarely” and “never”. Answers were grouped as dichotomous to exposed and unexposed groups: the exposed group included respondents who answered “rather often”, “sometimes” or “rarely” while the unexposed group reported “never”. Respondents who answered “I don’t know/hard to say” were considered as missing values and excluded from the analysis. The number of people included in each analysis is specified in the result tables.

#### 2.2.3. Confounding Variables

The following confounding variables were included in the regression models: gender, age, education and year of the survey. Age was divided into the following groups: 18–24, 25–34, 35–44, 45–54, 55–63 and 64–80. The education level was determined as preschool or incomplete primary, primary, secondary, vocational secondary or higher education. 

Job levels were merged and categorised as head of the company, senior or middle manager; senior and intermediate level specialist; service and sales employee; skilled worker; unskilled worker. The length of work experience with the current employer was measured in the following periods: less than 1 month, less than 6 months, 6 months to 1 year, 1 to 2 years, 2 to 5 years, 5 to 10 years, more than 10 years. For data analysis purposes the periods of the length of work were re-grouped into the following categories: less than 1 year, 1 to 5 years, 5 to 10 years, 10 years and more. 

Different income categories were used in surveys between 2006 and 2018. In addition, the currency changed from the Latvian Lat to Euros in 2014. For data analysis purposes the respondents were divided into groups according to income quartiles based on their monthly individual salary reports in each survey year.

### 2.3. Statistical Analysis

Survey data from 2006 to 2018 were merged to create a dataset for the statistical analysis. Descriptive analyses (mean, standard deviation) and frequency analyses (percentages, distribution) were used to describe the data. The association between conflicts at work and sickness absence was analysed by using binomial logistic regression and calculated as odds ratios (ORs) with 95% confidence intervals (CIs) in adjustment to gender, age, education and survey year. 

Interactions between conflicts at the workplace and the survey year were tested in association with sickness absence. Since no significant interactions were found, the interaction term was not included in the final regression models. 

The Spearman correlation coefficient was calculated to check multicollinearity between age and work experience and no significant multicollinearity was found. 

The analysis was conducted using the IBM SPSS Statistics 26 (IBM Corporation, Armonk, New York, NY, USA) software.

## 3. Results

Medically certified sickness absence was reported by 33.8% (*n* = 2891) of all respondents. The prevalence of sickness absence in socio-demographic groups is presented in Table A2. The unadjusted odds of sickness absence increased by 22% in females in comparison to males. The odds of sickness absence were significantly higher among 25–44-year-old respondents compared to the youngest age group, but no statistical differences were found in other age groups. Work experience of less than 1 year with the current employer significantly decreased the odds of sickness absence, but there were no significant associations with other work experience periods. The level of education was not associated with sickness absence, but the odds of sickness absence increased along with higher salaries, also after adjustment for other socio-demographic factors. Unadjusted odds of sickness absence decreased equally among both higher-level job holders and unskilled workers compared with intermediate-level job holders. However, after adjustment for other socio-demographic factors, the odds of sickness absence also decreased for service and sales employees but increased for skilled workers (Table 2).

The most reported types of conflict at work were conflicts between managers and employees (54.2%) followed by conflicts between employees (44.8%) and with customers (41.6%). Conflicts between groups of employees were less prevalent (26.3%) (Table A3).

The odds of conflicts between managers and employees were significantly higher in males, but conflicts with customers were found to be more frequent in females. In terms of conflicts between employees as well as between groups of employees there was no difference between genders.

In general, the odds of all of the studied types of workplace conflict decreased with age. Work experience of less than 1 year with the current employer was linked with significantly lower odds of workplace conflicts. Having a higher education, higher salary and holding a higher position (heads and managers) were associated with higher odds of conflicts at the workplace (Table 3).

The odds of sickness absence were significantly increased across all types of conflict at work. Among the respondents who reported conflicts between managers and employees or between employees and groups of employees, the odds of sickness absence increased by 39–51% after adjustment for socio-demographic factors. A weaker, but still significant association was found between sickness absence and having conflicts with customers (Table 4).

## 4. Discussion

In general, this study shows that employees who face any type of workplace conflict have significantly higher odds of sickness absence. The associations remained strong and did not change substantially after adjusting for gender, age, educational level and survey year.

We found that, on average, 1 in 3 respondents reported medically certified sickness absence within the previous year. Those self-reported rates are slightly higher than the average sickness absence rates in the European Union (EU)—28% of employees said they had been absent for health reasons for 5 or more days in the last 12 months [37]. Our study shows that the prevalence of sickness absence is higher among female employees which is also consistent with findings of other studies from Western countries [38,39]. Data from the literature show that there are well-studied factors (e.g., health, job control/demands) and less-studied factors (e.g., parent–child conflict and sexual assault) that partly explain the gender gap in Western countries; however, it remains mostly unexplained [39]. When it comes to Latvia, we would like to stress that women are better at looking after their health than men. They visit physicians more often and report both physical and mental health concerns to their doctors [3]. These explanations are also consistent with other countries [40,41]. Increased sickness absence among female workers can be caused by psychosocial work factors: exposure to emotional demands, pressure to hide emotions, low degree of freedom, low quality of leadership and low job promotion rates [38]. However, it can also be partly explained with conflicts with customers. Therefore, it is likely that actions taken by employers to reduce such types of conflict will influence the rate of sickness absence on a company level.

Our results on the reporting of sickness absence show that younger employees report more sickness absence than older ones. Although the prevalence of illnesses increases with age, sickness absence is a more complex phenomenon than the direct correlation between age and illness [7]. Previous studies suggest that older workers may face more difficulties to find a new job in case of job loss due to health problems, so they avoid sickness absence. However, younger employees potentially have different motivations to work than older workers [42]. Decreased sickness absence in the 63+ (above retirement age) age group can be explained by healthy worker bias, as those who are not healthy are most likely to retire. Our results do not support previously published results that older people may be more likely to become sick in case of conflicts at the workplace. The results of our study show that all types of workplace conflict were significantly less reported by employees in the 55+ age group. Conflicts between managers and employees were more frequent in the 25–34 and 35–44 age groups. All types of conflict decrease with age, but, if we compare the odds in age groups over 45, conflicts with customers are more common than other types of conflict. This means that, when focusing on workplace interventions, employers should focus on younger employees for all types of interventions, for conflicts between managers and employees—on all employees under the age of 45, and for conflicts with customers on all those above 45. This might also mean that conflict management skills should be improved at the secondary and higher education level, as these skills are useful in all aspects of life, e.g., working life, family.

It has been previously reported that sickness absence rates are lower among professionals, the higher educated and employees with higher income [43]; however, based on our results, we can only partly agree with this statement. We did not identify any significant difference in sickness absence among employees with different levels of education. However, management-level employees have reported less sickness absence which can be explained by higher income enabling people to make healthier decisions in everyday life [26,43]. Some employees need to work while being sick due to the salary system (e.g., no benefits for sickness absence if no taxes are paid, in the case of the piece-rate wage system) [3]. In addition, the national social security system has an impact on sickness absence culture, especially on employees with lower income levels. They tend not to take sick leave because Latvian legislation states that the first day of sickness absence is not paid. From the 2nd to the 10th day of illness the employer pays sickness absence pay (75% of your average earnings). From the 11th day of illness, the State Social Insurance Agency grants a sickness benefit (at the rate of 80% of the average salary). That means that income during periods of sickness is lower. This most affects employees with the lowest income and plays an important role in the decision of whether to take sick leave or not. These decisions can explain why unskilled workers who typically also belong to lower income workers report low sickness absence levels. In addition, workers from management level and unskilled workers have different work-related physical and mental risk factors which might result in different underlying causes for sickness absence—for managers, it is more likely to be related to demanding work, overload, burnout, etc., but less qualified workers suffer from physical diseases [43].

When looking at the results of our study in terms of genders having different types of workplace conflicts, there are two major differences in the prevalence of conflicts between female and male employees. If the risk for having conflicts with customers is significantly higher for females, then the risk for conflicts between managers and employees is higher for males. This can be explained by several aspects—women are more relationship-oriented than men and more attuned to relationships with others, which might be the reason for fewer conflicts with persons from the same company [44]. Besides, there already exists evidence that different jobs have different types of conflicts [45], and women are more likely to work in customer service, the service sector, or other jobs where they have direct contact with people who are not co-workers in their workplace (e.g., healthcare, education) and there is an excessive need to hide emotions in conflicts with customers [38].

The strongest association in this study was found between sickness absence and conflicts between managers and employees, which is consistent with published data on conflict management. It has been suggested that, if workers perceive that managers handle conflicts in an integrative way, they feel more committed to the organization and therefore will tend to have less sickness absence [33]. In addition, an association between poor leadership and higher risk of more sickness absence days among employees [10,46,47,48] has been reported. Autocratic leadership (presumably in the presence of conflicts) has also been related to a greater amount of total sick days [46]. As conflicts with management and conflicts with co-workers can impact the organisational climate, fairness and role clarity, those can lead to a remarkable increase in sickness absence. An increased risk of sickness absence can be also explained by emotional dissonance which increases the feeling of being exhausted [49,50,51]. Although typically these findings come from studies on employees who interact with customers where managing emotions is a required skill [51,52,53], employees are also likely to need to regulate their feelings in conflicts with management. This can at least partly explain the increased odds for sickness absence in the case of conflicts between managers and employees. Managers themselves can be the risk group for conflicts between managers and employees as it was found that the odds of conflicts between managers and employees significantly increased for those with the highest-level jobs. In addition, managers have an important moderating role in the management of other types of conflict at work [54]. However, this group was not homogenous and head and middle managers were classified as a single group. In this study, the association between sickness absence and conflicts at the workplace was not adjusted for job position; however, further studies might explore the differences among groups holding different job positions.

Key approaches to sickness absence management (e.g., focused attendance management, integrated disability management, benefit design, workplace health promotion, wellbeing programs) as well as stress reduction (e.g., staff training on customer relations) might not be sufficient, and attention is needed on building a present and committed working environment [33]. Based on the results of the association, it was possible to identify the possible risk factors leading to increased sickness absence and interventions to be taken by the employer. This explored the role of psychosocial work factors, but especially that of different conflicts at work on sickness absence and may be useful to better prevent this outcome if the issue is brought up from an individual to an organisational level [55]. In this context, workplace interventions should include integrative conflict management strategies, the improvement of performance evaluation, management development or training etc.

We have identified several limitations of this study. One of the limitations is missing data on the length of sickness absence and number of cases per year as that would give the opportunity to better capture the complexity of sickness absence. Still, studies on the agreement between the annual number of self-reported and officially recorded sickness absence suggest that data matching is relatively good and that associations with health are equivalent for both measures [56].

Another limitation of our study was a lack of information on the respondents’ state of health (e.g., overall state or whether they are suffering from any chronic disease) which made it impossible to adjust the data according to the state of health. Recall bias answering questions on sickness absence within the previous year may be present. However, this study included a large sample of respondents which is representative of the total working population in Latvia.

## 5. Conclusions

A better understanding of sickness absence and its non-medical causes is important for identifying the most appropriate workplace interventions. That would result in fewer costs to employers, governments and workers, which is extremely important because effective sector-specific measures to be implemented at an organisational level are available for reducing the number of conflicts at work.

The chance of sickness absence resulting from conflicts between managers and employees is higher than that resulting from other types of workplace conflict. This leads to the conclusion that improving managers’ leadership and human resource management skills should be a priority in building committed working environments.

On a policy level, when planning measures to reduce sickness absence rates among employees, attention should be given to effective conflict management as part of developing and maintaining a health-promoting workplace.

## Figures and Tables

**Table 1:** Description of the Work conditions and risks in Latvia study sample.

Survey	Total Number of Study Population	Number of Respondents Included in Analyses
2006	2520	2118
2010	2505	2065
2013	2558	2066
2020	2501	2308
Total	10,084	8557

**Table 2:** The odds of self-reported medically certified sickness absence within the previous year in association with socio-demographic factors.

	Certified Sickness Absence, OR (CI 95%) ^a^, Unadjusted	Certified Sickness Absence, OR (CI 95%) ^a^, Adjusted for Gender, Age, Education and Survey Year	
Gender			
Female	1.22 * (1.12–1.34)	1.24 * (1.13–1.35)	
Male	1	1	
Age			
64–80 years	0.85 (0.63–1.14)	0.83 (0.61–1.11)	
55–63 years	1.12 (0.92–1.37)	1.06 (0.87–1.29)	
45–54 years	1.14 (0.95–1.37)	1.10 (0.91–1.32)	
35–44 years	1.27 ** (1.06-1.53)	1.23 *** (1.03–1.48)	
25–34 years	1.34 ** (1.12–1.62)	1.31 ** (1.09–1.58)	
18–24 years	1	1	
Education			
Preschool or incomplete primary education	0.99 (0.54–1.84)	1.07 (0.58–1.98)	
Primary education	0.93 (0.77–1.11)	0.99 (0.82–1.19)	
Secondary education	0.94 0.83–1.07)	0.99 (0.87–1.12)	
Vocational secondary education	0.93 (0.83–1.07)	0.98 (0.88–1.09)	
Higher education		1	1
Salary			
1st quartile (lowest)		1	1
2nd quartile		1.31 * (1.13–1.52)	1.33 * (1.15–1.55)
3rd quartile		1.33 * (1.16–1.54)	1.39 * (1.20–1.61)
4th quartile (highest)		1.41 * (1.23–1.62)	1.56 * (1.34–1.81)
Job category			
Head of the company, senior manager or middle manager		0.76 ** (0.64–0.92)	0.80 *** (0.67–0.97)
Senior and intermediate level specialist		1	1
Service and sales employee		0.89 (0.78–1.02)	0.86 *** (0.75–0.99)
Skilled worker		1.03 (0.92–1.15)	1.15 *** (1.01–1.31)
Unskilled worker		0.78 ** (0.67–0.91)	0.80 *** (0.67–0.95)
Work experience with current employer			
Less than 1 year		0.56 * (0.48–0.65)	0.54 * (0.46–0.63)
1 to 5 years		1	1
5 to 10 years		1.02 (0.91–1.15)	1.08 (0.96–1.23)
10 years and more		0.98 (0.87–1.09)	1.08 (0.95–1.21)

^a^. The reference category for sickness absence group is group of respondents, who did not fall ill previous year. * *p* < 0.001. ** *p* < 0.01. *** *p* < 0.05.

**Table 3:** Unadjusted odds of conflicts at work in association with the socio-demographic factors of respondents.

	Conflicts between Managers and Employees, OR (CI 95%) ^a^	Conflicts between Employees, OR (CI 95%) ^a^	Conflicts between Groups of Employees, OR (CI 95%) ^a^	Conflicts with Customers, OR (CI 95%) ^a^
Gender				
Female	0.79 * (0.72–0.86)	1.05 (0.96–1.14)	1.01 (0.91–1.11)	1.65 * (1.51–1.81)
Male	1	1	1	1
Age				
64–80 years	0.39 * (0.29–0.52)	0.38 * (0.28–0.50)	0.42 * (0.30–0.60)	0.56 * (0.42–0.75)
55–63 years	0.66 * (0.55–0.79)	0.51 * (0.42–0.61)	0.52 * (0.42–0.64)	0.64 * (0.54–078)
45–54 years	0.99 (0.83–1.17)	0.71 * (0.60–0.85)	0.70 * (0.58–0.84)	0.77 ** (0.65–0.91)
35–44 years	1.27 ** (1.07–1.50)	0.94 (0.80–1.12)	0.99 (0.82–1.19)	1.05 (0.89–1.26)
25–34 years	1.22 *** (1.03–1.46)	1.05 (0.88–1.24)	1.12 (0.93–1.36)	1.10 (0.92–1.32)
18–24 years	1	1	1	1
Education				
Preschool or incomplete primary education	0.68 (0.37–1.23)	0.74 (0.41–1.34)	0.95 (0.50–1.79)	0.32 * (0.17–0.63)
Primary education	0.59 * (0.49–0.70)	0.61 * (0.51–0.73)	0.60 * (0.49–0.74)	0.25 * (0.21–0.31)
Secondary education	0.71 * (0.62–0.80)	0.63 * (0.56–0.71)	0.56 * (0.49–0.65)	0.46 * (0.40–0.52)
Vocational secondary education	0.78 * (0.70–0.86)	0.68 * (0.62–0.76)	0.65 * (0.58–0.72)	0.53 * (0.47–0.58)
Higher education	1	1	1	1
Salary				
1st quartile (lowest)	1	1	1	1
2nd quartile	1.26 ** (1.09–1.45)	1.36 * (1.18–1.58)	1.11 (0.94–1.32)	1.20 *** (1.03–1.40)
3rd quartile	1.38 * (1.21–1.58)	1.50 * (1.31–1.71)	1.34 * (1.14–1.57)	1.47 * (1.28–1.68)
4th quartile (highest)	1.82 * (1.59–2.07)	1.98 * (1.74–2.26)	1.77 * (1.52–2.07)	1.70 * (1.49–1.95)
Position				
Head of the company, senior manager or middle manager	1.39 * (1.16–1.66)	1.50 * (1.26–1.78)	1.24 *** (1.03–1.49)	1.21 *** (1.02–1.44)
Senior and intermediate level specialist	1	1	1	1
Service and sales employee	0.84 ** (0.74–0.96)	0.81 * (0.71–0.92)	0.70 * (0.60–0.81)	1.57 * (1.38–1.79)
Skilled worker	0.93 (0.84–1.04)	0.79 * (0.71–0.88)	0.75 * (0.66–0.85)	0.35 * (0.31–0.39)
Unskilled worker	0.48 * (0.42–0.56)	0.59 * (0.51–0.68)	0.55 * (0.46–0.66)	0.26 * (0.22–0.32)
Work experience				
Less than 1 year	0.64 * (0.56–0.74)	0.71 * (0.61–0.81)	0.75 * (0.63–0.88)	0.70 * (0.60–0.81)
1 to 5 years	1	1	1	1
5 to 10 years	1.10 (0.98–1.25)	1.01 (0.89–1.13)	1.09 (0.96–1.25)	1.00 (0.88–1.13)
10 years and more	0.99 (0.89–1.11)	1.05 (0.95–1.17)	1.01 (0.89–1.14)	0.98 (0.88–1.09)

^a^. The reference category is respondents who have no conflicts. * *p* < 0.001, ** *p* < 0.01, *** *p* < 0.05.

**Table 4:** The odds of self-reported medically certified sickness absence within the previous year in association with conflicts at work.

	Certified Sickness Absence, *n* (%)	Certified Sickness Absence, OR (CI 95%) ^a^, Unadjusted	Certified Sickness Absence, OR (CI 95%) ^a^, Adjusted for Gender, Age, Education and Survey Year
Conflicts between managers and employees	2801		
Yes	1693 (60.4)	1.47 * (1.34–1.61)	1.51 * (1.37–1.66)
No	118 (39.6)	1	1
Conflicts between employees	2865		
Yes	1436 (50.1)	1.39 * (1.27–1.52)	1.39 * (1.27–1.52)
No	1429 (49.9)	1	1
Conflicts between groups of employees	2786		
Yes	867 (31.1)	1.45 * (1.31–1.60)	1.45 * (1.31–1.61)
No	1919 (68.9)	1	1
Conflicts with customers	2709		
Yes	1186 (43.8)	1.14 ** (1.04–1.25)	1.11 *** (1.01–1.23)
No	523 (56.2)	1	1

^a^. The reference category for sickness absence group is group of respondents, who did not fall ill previous year. * *p* < 0.001, ** *p* < 0.01, *** *p* < 0.05.

## Data Availability

The original datasets of the independent studies Work conditions and risks in Latvia are available upon an official data request from the relevant data owners: 2006—from the Ministry of Welfare (https://www.lm.gov.lv/en), 2010 and 2013—from the Employers’ Confederation of Latvia (https://lddk.lv/en/), 2020—from the State Labour Inspectorate (https://www.vdi.gov.lv/en).

## Appendix A

**Table A1:** Socio-demographic characteristics of the study sample.

Distribution of the Total Study Sample, *n* (%)	
Gender	
Female	4601 (53.8)
Male	3956 (46.2)
Age	
64–80 years	313 (3.7)
55–63 years	1458 (17.0)
45–54 years	2218 (25.9)
35–44 years	2080 (24.3)
25–34 years	1759 (20.6)
18–24 years	727 (8.5)
Education	
Primary school or incomplete elementary education	46 (0.5)
Elementary school education	652 (7.6)
Secondary school education	1814 (21.2)
Vocational secondary education	3468 (40.5)
Higher education	2577 (30.1)
Salary	
1st quartile (lowest)	1536 (19.6)
2nd quartile	1645 (21.0)
3rd quartile	2229 (28.4)
4th quartile (highest)	2434 (31.0)
Position	
Head of the company, senior or mid—level manager	668 (7.8)
Senior and intermediate level specialist	2682 (31.5)
Service and sales worker	1449 (17.0)
Skilled worker and craftsman	2681 (31.5)
Unskilled worker	1035 (12.2)
Work experience	
Less than 1 year	1191 (14.0)
1 to 5 years	2991 (35.3)
5 to 10 years	1735 (20.5)
10 years and more	2563 (30.2)

**Table A2:** Prevalence of self-reported medically certified sickness absence by socio-demographic factors, *n* (%).

	Had Reported Sickness Absence	Had not Reported Sickness Absence
Gender		
Female	1650 (35.9)	2951 (64.1)
Male	1241 (31.4)	2715 (68.6)
Age		
64–80 years	84 (26.8)	229 (73.2)
55–63 years	474 (32.5)	984 (67.5)
45–54 years	731 (33.0)	1487 (67.0)
35–44 years	737 (35.4)	1343 (64.6)
25–34 years	645 (36.7)	1114 (63.3)
18–24 years	219 (30.1)	508 (69.9)
Education		
Preschool or incomplete primary education	16 (34.8)	30 (65.2)
Primary education	216 (33.1)	436 (66.9)
Secondary education	609 (33.6)	1205 (66.4)
Vocational secondary education	1151 (33.2)	2317 (66.8)
Higher education	899 (34.9)	1678 (65.1)
Salary		
1st quartile (lowest)	443 (28.8)	1093 (71.2)
2nd quartile	571 (34.7)	1074 (65.3)
3rd quartile	782 (35.1)	1447 (64.9)
4th quartile (highest)	885 (36.4)	1549 (63.6)
Job category		
Head of the company, senior manager or middle manager	195 (29.2)	473 (70.8)
Senior and intermediate level specialist	940 (35.0)	1742 (65.0)
Service and sales employee	471 (32.5)	978 (67.5)
Skilled worker	955 (35.6)	1726 (64.4)
Unskilled worker	307 (29.7)	728 (70.3)
Work experience with current employer		
Less than 1 year	280 (23.5)	911 (76.5)
1 to 5 years	1066 (35.6)	1925 (64.4)
5 to 10 years	628 (36.2)	1107 (63.8)
10 years and more	899 (35.1)	1664 (64.9)

**Table A3:** The distribution of the studied types of conflict at the workplace in the sample, *n* (%).

	Type of Conflict at Workplace	*n*, (%)
Conflicts between managers and employees	Yes	4483 (54.2)
	No	3794 (45.8)
Conflicts between employees	Yes	3785 (44.8)
	No	4669 (55.2)
Conflicts between groups of employees	Yes	2155 (26.3)
	No	6050 (73.7)
Conflicts with customers	Yes	3367 (41.6)
	No	4669 (55.2)
